# Highly Sensitive and Specific Panels of Plasma Exosomal microRNAs for Identification of Malignant Pulmonary Nodules

**DOI:** 10.1111/crj.70034

**Published:** 2024-11-15

**Authors:** Rui Tao, Dandan Wang, Wenjing Pei, Yanfei Liu, Pengcheng Liu, Renming Li, Jiegou Xu, Jing Ye, Dahai Zhao

**Affiliations:** ^1^ Department of Respiratory and Critical Care Medicine, the Second Affiliated Hospital Anhui Medical University Hefei Anhui Province China; ^2^ Department of Immunology, School of Basic Medical Sciences Anhui Medical University Hefei Anhui Province China; ^3^ Department of Respiratory and Critical Care Medicine, Anhui Chest Hospital Thoracic Clinical College of Anhui Medical University Hefei Anhui Province China

**Keywords:** diagnostic biomarkers, early diagnosis, exosomal microRNAs, plasma, solitary pulmonary nodules

## Abstract

**Objectives:**

With wide application of computed tomography (CT) in early lung cancer screening, solitary pulmonary nodules (SPNs) are frequently detected. Due to their high etiological diversity and potential for malignancy, rapid and accurate identification and malignant SPNs are crucial in the clinical management. In the present study, plasma exosomal microRNAs were identified and evaluated as sensitive and specific indicators for malignant SPNs.

**Materials and Methods:**

Exosomal miRNAs isolated from the plasmas of pathologically confirmed patients with SPN (four malignant and four benign, designated as the screening set) were subjected for high throughput sequencing and eight candidate miRNAs were selected. The pre‐operation plasma levels of the candidate miRNAs in 77 patients with SPN (48 malignant and 29 benign, designated as the identification set) were detected by quantitative PCR, five miRNAs were identified as potential biomarkers for malignant SPNs, and the diagnostic values of the five miRNAs each alone or combined were then analyzed by AUROC analysis. The prediction values of the identified miRNAs were further evaluated in 95 patients with SPN (double blind, 74 malignant and 21 benign, designated as the validation set).

**Results:**

High‐throughput sequencing identified 45 miRNAs with statistical differences between benign and malignant SPNs. Among the eight candidate miRNAs in the identification set, miR‐1‐3p alone had the best diagnostic value, with the sensitivities and specificities of 89.6% and 100% for malignant SPNs. Unexpectedly, when miR‐1‐3p was combined with miR‐99a‐5p, both the sensitivity and specificity reached 100% for malignant SPNs. miR‐1‐3p+miR‐125b‐5p and miR‐1‐3p+miR‐218‐5p were also good indicators of malignant SPNs with sensitivities of 95.8% and 97.9%, specificities of 100% and 96.6%. Further analysis of these microRNA combinations in the validation set indicated that the PPV were 91.4%, 97.4%, and 93.5% and the NPV were 100%, 100%, and 88.9% for miR‐1‐3p+miR‐99a‐5p, miR‐1‐3p+miR‐218‐5p, and miR‐1‐3p+miR‐125b‐5p, with the sensitivities were 100%, 100%, and 97.3% and the specificities were 66.7%, 90.5%, and 76.2% for miR‐1‐3p+miR‐99a‐5p, miR‐1‐3p+miR‐218‐5p, and miR‐1‐3p+miR‐125b‐5p, respectively.

**Conclusions:**

Through high throughput sequencing, qPCR determination of plasma microRNAs and AUROC analysis, miR‐1‐3p combined with miR‐99a‐5p, miR‐125b‐5p, or miR‐218‐5p have been found to be sensitive and specific indicators of malignant SPNs in both the identification and validation sets. Our results indicate that the panels of plasma miRNAs can be used as diagnostic biomarkers for malignant SPNs.

## Graphical abstract

Through high throughput sequencing, qPCR determination of plasma microRNAs and AUROC analysis, miR‐1‐3p combined with miR‐99a‐5p, miR‐125b‐5p, or miR‐218‐5p have been found to be sensitive and specific indicators of malignant SPNs. Our results indicate that the panels of plasma miRNAs are potential diagnostic biomarkers for malignant SPNs.

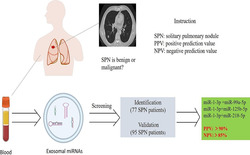

## 1 Introduction

The early stage of lung cancer (especially non‐small cell lung cancer) is mainly in the form of small nodules in the lungs, and the detection rate of pulmonary nodules in healthy population has been greatly improved with comprehensive application of low‐dose computed tomography (CT) screening in recent years. Solitary pulmonary nodules (SPNs) represent solitary round lesions with diameter of less than 3.0 cm on CT scan, which are generally not accompanied by atelectasis, lymphadenopathy, and pleural effusion [1, 2]. But pulmonary nodules are not the specific manifestations of early lung cancer, most pulmonary nodules are benign lesions, including granuloma (inflammatory nodules), tuberculosis infection and fungal infections such as spore bacteria. Pulmonary nodules can also occur in immune‐related diseases including rheumatoid arthritis and granulomatosis with polyangiitis [3, 4]. The US National Lung Cancer Screening Test showed a 24.2% positive screening rate for pulmonary nodules among healthy people, but 96.4% were benign [5]. Thus, judging whether pulmonary nodules are malignant or not is crucial in the clinical management. Although CT findings including nodule size, lobar location, density and margin characteristics can help the judgment, there are still 30% to 50% of the nodules are benign after surgical operation [6]. Nevertheless, long‐term follow‐up observation with CT increases economical and mental burden of the patients. Traditional serological tumor markers such as neuron‐specific enolase, carcinoembryonic antigen and squamous cell carcinoma antigen are limited in differentiating malignant from benign pulmonary nodules because of the very early stage of lung cancer [7]. Since the sizes of pulmonary nodules are usually small, accurate sampling for tissue biopsy is difficult. Also, tissue biopsy is invasive, and patients with other diseases such as severe emphysema and coagulation disorders, for example, are not suitable for the operation. Therefore, it is important to find novel biomarkers with high specificity and sensitivity for early diagnosis and accurate treatment of pulmonary nodules.

In recent years, plasma or serum biomarkers including cell‐free DNA, circulating tumor cells and microRNAs (miRNA), so called as liquid biopsies, have been extensively investigated for the diagnosis of pulmonary nodules. Among these biomarkers, plasma exosomal miRNAs shows greater potentials. MiRNAs are non‐coding, short, single‐stranded RNA with an average size of 22 nucleotides, and involved in the pathophysiological processes of the organism by regulating gene transcription and translation [8]. Circulating miRNAs are released from tumor cells and/or from tumor microenvironment, mostly packed into exosomes or associated with protein complexes [9], and represent some characteristics of the original cancer. Exosomes are small extracellular vesicles with the diameter of 40–100 nm, containing a variety of bioactive molecules, including proteins, nucleic acids (miRNA and lncRNA), and lipids, and participate in many pathological and physiological processes as intercellular communication signals [10]. As exosomes exist widely in various body fluids, their packed miRNAs can be extracted and detected as biomarkers for early cancer detection [11, 12, 13]. Studies have shown exosomal miRNAs as potential biomarkers for lung cancer diagnosis, with an average sensitivity of 83% and a specificity of 84% [14, 15, 16, 17]. In the diagnosis of SPN, a recent study has indicated that exosomal miR‐185‐5p/miR‐32‐5p and miR‐140‐3p/let‐7f‐5p in the plasma display promising diagnostic values, with a sensitivity (59.3% and 85.1%) and specificity (75% and 100%) [18].

To date, accumulating data suggest that exosomal miRNAs could aid in the diagnosis of pulmonary nodules, but their sensitivities and specificities should be increased, especially for the small SPNs with diameter of about 1 cm or smaller. In this study, to find out novel panels of exosomal miRNAs, we first searched candidate plasma exosomal miRNAs in pathologically confirmed patients with SPN by high throughput sequencing, then detected their plasma levels by quantitative PCR in pathologically confirmed patients with SPN in a large scale for analysis of the diagnostic values of the candidate miRNAs each alone or combined, and finally blindly evaluated the prediction values in patients with SPN. Three panels of miRNAs were identified as sensitive and specific indicators of malignant SPNs.

## 2 Materials and Methods

### 2.1 Patients and Study Design

Patients with SPNs in Department of Cardiothoracic Surgery and Department of Respiratory and Critical Care Medicine, the Second Affiliated Hospital of Anhui Medical University from September 2019 to June 2021 were included in this study. The inclusion criteria for participants were as follows: 1) Patients were diagnosed of pulmonary nodules by CT imaging conformity without associated respiratory symptoms; 2) Patients had not received any surgery, radiotherapy, chemotherapy and other anti‐tumor treatments before being included in the study; 3) Pathological examination of surgically removed SPN tissues was performed after hospitalization; 4) Patients without a history of lung cancer and other tumors. Patients' clinical data and blood samples were collected before the surgical operation, and the pathological results were collected after the operation. We did not calculate the sample size, since it is hard to determine the sample size of the benign and malignant SPNs group by power calculations on the basis of the population incidence of SPNs and the probability of benign or malignant SPNs.

To find specific miRNAs for identification of benign or malignant pulmonary nodules, included patients with SPN were grouped in three sets: the screening set (miRNA screening with high‐throughput sequencing of plasma exosomal miRNAs), the identification set (potential miRNA biomarkers selection with qPCR and AUROC) and the validation set (further evaluation of the prediction values*)*. The screening set was comprised of four benign (two nonspecific inflammation and two tuberculosis) and four malignant (two adenocarcinomas in situ and two minimally invasive adenocarcinomas) patients with SPN determined by pathological examination of the surgically resected tissues. The age‐and sex‐matched patients (approximate 50 years old, two males and two females in the benign and malignant groups) were chosen for that their histological types were the most common types of benign and malignant SPNs. Patients with SPN with serious diseases in the major organs were excluded given that these diseases may affect miRNA generation, including infectious diseases such as EBV, HIV, HCV, various bacterial infections and non‐infectious diseases such as autoimmune diseases, metabolic disorders, and genetic diseases. The pre‐operation plasma samples of the included patients with SPN were subjected for isolation of exosomal miRNAs and high‐throughput sequencing to search for candidate miRNAs. The identification set of patients with SPN who were admitted to the hospital from September 2019 to May 2020 and met the inclusion criteria contained a total of 77 patients with SPN. Their plasma levels of the candidate miRNAs were detected by quantitative polymerase chain reaction (qPCR), the candidate miRNAs with statistical difference were identified as potential biomarkers, and the diagnostic values of the potential miRNA biomarkers each alone or combined were evaluated by area under the receiver operating characteristic (AUROC). The validation set of 95 patients with SPN were admitted to the hospital from June 2020 to June 2021 and met the inclusion criteria. Their plasma levels of the potential miRNAs were subjected to analyze the positive predictive value (PPV), negative predictive value (NPV) and other parameters in a blinded fashion by using the optimal critical values established in the identification set.

### 2.2 Plasma Exosomal microRNA Isolation

Five milliliters of blood samples from patients with SPN were collected in vacutainer tubes with EDTA, centrifugated at 1500 ×*g* for 15 min at 4 °C within 1 h, and 2 mL of the plasma samples were collected and stored at −80 °C before use. The exosomal miRNAs were extracted using the exoRNeasy Serum/Plasma Midi Kit (Qiagen, Hilden, Germany) in accordance with the manufacturer's protocol. Total miRNA was eluted in 14 μL ddH_2_O, and after determination of miRNA concentration, each miRNA sample was kept at −80 °C until use.

### 2.3 High‐Throughput Sequencing of Plasma Exosomal miRNAs and Candidate miRNA Screening

Eight miRNA samples isolated from the screening set of patients with SPN (four malignant, and four benign) were subjected for high‐throughput sequencing by using Illumina Hiseq 3000 (Genergy Bio‐Technology, Shanghai, China). Adaptor‐, N base‐and poly A/T‐ containing reads of total raw sequencing reads were filtered, and low‐quality reads and the reads exceeding the length limit (shorter than 15 bases, longer than 40 bases) were trimmed by Fastx_toolkit (0.0.14) to obtain clean reads. The quality control of the clean reads was assessed using Seqtk (1.0‐r82‐dirty), and the clean reads were aligned to the human genome, Rfam database, RepBase and miRBase by Bowtie (1.0.0). miRNA expression was normalized as counts per million (CPM) and differentially expressed exosomal miRNAs between benign and malignant SPNs were analysed by DESeq. The mean CPM of each miRNA within‐group was calculated and the difference between the groups was calculated as log2 (foldchange) and *p* ≤  0.05 was considered to be statistically significant.

Candidate miRNAs was first screened by p value and log2 foldchange. Only the miRNAs with *p* ≤  0.05 and log2 foldchange ≤ −1 or ≥ 1 were selected. The selected miRNAs were then screened by adjusted p values, which were calculated by the Benjamini and Hochberg procedure to control the false discovery rate (FDR). Only the miRNAs with adjusted *p* value ≤ 0.05 were chosen. Considering the small sample size, the candidate miRNAs were further selected by the following screening criteria: the CPM of an miRNA did not overlap, i.e., the CPM of any sample in one group was higher or lower than any sample in the other group.

### 2.4 Reverse Transcription of miRNAs

Two microliters of the isolated plasma exosomal miRNA samples were used for reverse transcription with Hairpin‐it™ microRNA and U6 snRNA normalization RT‐PCR Quantitation Kit (GenePharma, Shanghai, China) according to the manufacturer's instruction. The reverse transcription primers were designed according to the candidate miRNA sequences. The total reaction volume for reverse transcription was 10 μL, including 5 × MMLV RT buffer 2 μL; dNTP 0.375 μL; miRNA RT primers 1.2 μL; RNasin 0.125 μL; MMLV reverse transcriptase 0.1 μL; miRNA Sample 2 μL; and add RNase‐free H_2_O to 10 μL reaction system. The reverse transcription reaction was proceeded at 25 °C for 15 min; 42 °C for 30 min; 85 °C for 5 min and terminated at 4 °C.

### 2.5 Quantitative PCR Analysis of Candidate miRNAs

After reverse transcription, the quantitation of the candidate miRNAs was performed by PCR using the Hairpin‐it™ microRNA and U6 snRNA normalization RT‐PCR Quantitation Kit (GenePharma, Shanghai) according to the manufacturer's instruction. Briefly, 2 μL of reverse transcription samples were added with 10 μL of 2 × Real‐time PCR master Mix, 0.4 μL of miRNA specific primer set, 0.2 μL of Taq DNA polymerase (5 U/μL), and finally, ddH_2_O to 20 μL reaction system. Duplicate samples were analyzed. MicroRNA mimics of the candidate miRNA (GenePharma, Shanghai) were used to establish a standard curve, and plasma concentration of each candidate miRNA was calculated by the standard curves.

The reaction conditions for quantitative PCR were 95 °C for 3 min; 40 cycles of 95 °C for 12 s and 62 °C for 40 s. The primers for qPCR were described as follows: forward primer TTAGATTTAATCCGACGGGCG and reverse primer TATGGT‐TGTTGACGACTGGTTGAC for miR‐4‐3607; forward primer ACTGATAAATCC‐CTGAGACCCTAAC and reverse primer TATGGTTTTGACGACTGTGTGAT for miR‐125b‐5p; forward primer GCCTTTGGTCCCCTTCAAC and reverse primer TATGCTTGTTCTCGTCTCTGTGTC for miR‐133a‐3p; forward primer GCCGAA‐TGGAATGTAAAGAAGT and reverse primer TATGGTTTTGACGACTGTGTGAT for miR‐1‐3p; forward primer CGATTCCATTTGTGCTTGATCT and reverse primer TATGGTTTTGACGACTGTGTGAT for miR‐218‐5p; forward primer CCTCCT‐ATTTCCAGCATCAGTG and reverse primer TATGCTTGTTCTCGTCTCTGTGTC for miR‐338‐3p; forward primer CATTACTAAACCCGTAGATCCGAT and reverse primer TATGGTTTTGACGACTGTGTGAT for miR‐99a‐5p; and forward primer AGCCTCTCTCCTCTTTGGTTATCT and reverse primer TATGGTTGTTCTGCT‐CTCTGTGTC for miR‐9‐5p.

### 2.6 Statistical Analysis

Statistical analyses were performed with the IBM SPSS Statistics 26.0. The Shapiro–Wilk test were used for normality test of quantitative data, while the Mann–Whitney U test was used for non‐normally distributed data analysis and the t‐test was used for normally distributed data. The abilities of plasma levels of candidate miRNAs each alone or combined to predict the nature of SPNs were assessed by using AUROC, and the Youden indices were used to confirm the optimal cutoff values and their best sensitivity and specificity. The prediction values of the miRNA combinations in the validation set were blindly validated by comparing the calculated results with final pathological diagnosis. *p*‐Values < 0.05 was considered to be statistically significant.

## 3 Results

### 3.1 Candidate miRNA Screening and Selection

**FIGURE 1:**
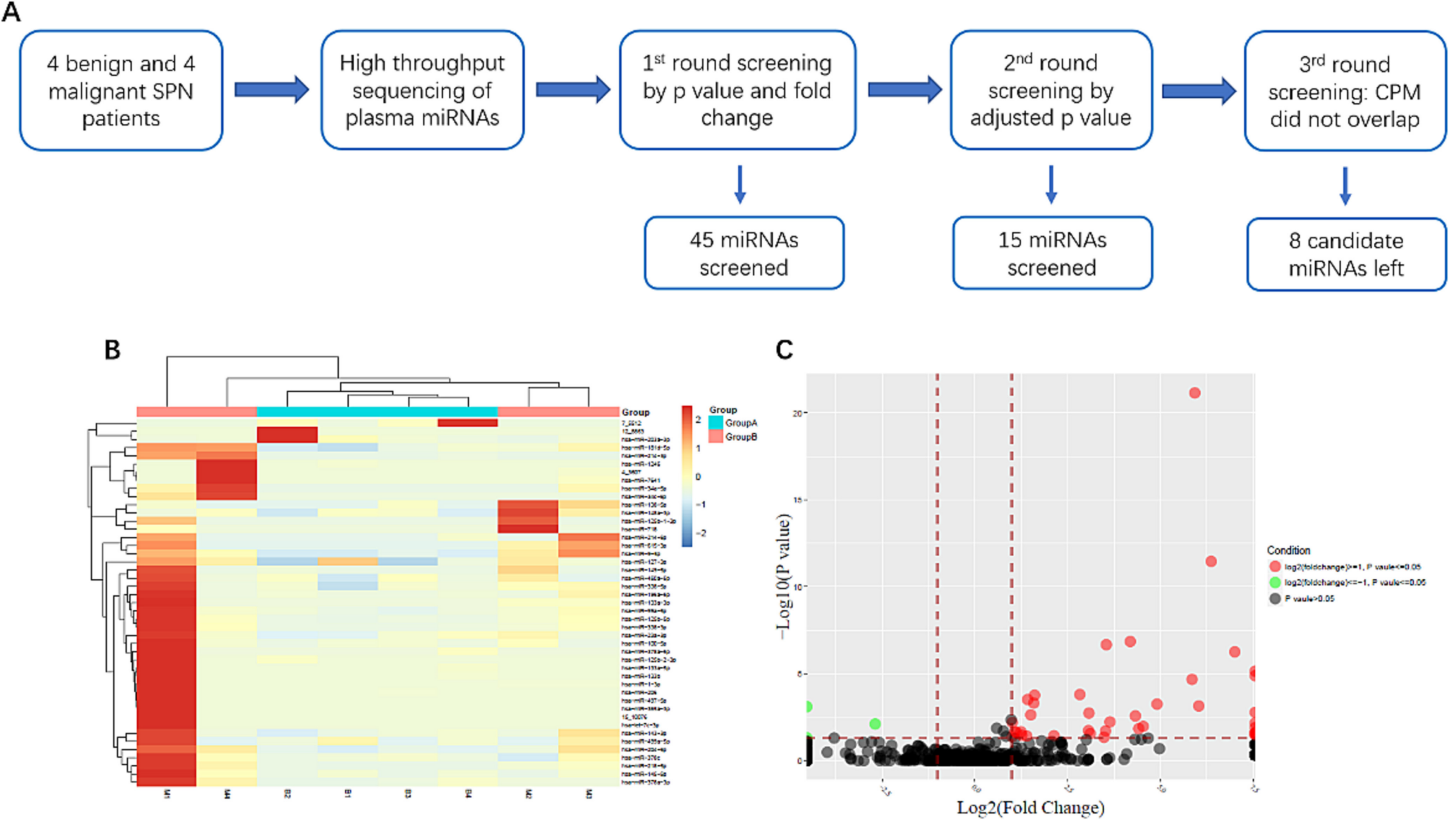
Screening of candidate miRNAs. A, flow chart for candidate miRNA screening; B, the heatmap of 45 differentially expressed mRNAs. The four columns (M1, M2, M3, and M4) and four columns (B1, B2, B3, and B4) represent the plasma miRNA samples from patients with malignant and benign SPN, respectively; C, volcano plot of 45 differentially expressed mRNAs.

For candidate miRNA screening, four patients with benign and four patients with malignant SPN were chosen and the SPNs were two nonspecific inflammation and two tuberculosis in the benign groups, and two adenocarcinomas in situ and two minimally invasive adenocarcinomas in the malignant groups, determined by pathological examination of the surgically resected tissues. Patients with benign and malignant SPN were age‐and sex‐matched patients, approximate 50 years old, two males and two females in each group. The histological types were the most common types representative of benign and malignant SPNs. Patients with SPN with serious diseases in the major organs were excluded given that these diseases may affect miRNA generation. The process for candidate miRNA screening is indicated in Figure 1A. First, plasma miRNA samples from malignant or benign SPNs were analyzed by high‐throughput sequencing, and 45 miRNAs were found to have statistical differences (*p* <  0.05) with log2 foldchange ≤ −1 or ≥ 1 (Figure 1B&C). Next, the 45 plasma exosomal miRNAs differentially expressed between malignant and benign SPNs were screened by adjusted p value, and the miRNAs with adjusted p values larger than 0.05 were trimmed. Fifteen miRNAs were reserved. Considering that the screening set had only eight patients with SPN and too many miRNAs to be analyzed in large scale, the 15 miRNAs were further screened by the following screening criterion: the CPM of any individual miRNA did not overlap, i.e., the CPM of any sample in one group was higher or lower than any sample in the other group. As shown in Table 1, eight candidate miRNAs including 4‐3607 (a novel microRNA), miR‐125b‐5p, miR‐133a‐3p, miR‐1‐3p, miR‐218‐5p, miR‐338‐3p, miR‐9‐5p and miR‐99a‐5p were initially selected.

**TABLE 1:** Candidate eight miRNAs selected by high‐throughput sequencing.

	CPM of benign SPNs (average)	CPM of malignant SPNs (average)	Fold change (Log2)	*p*	*p*.adj
**miR‐4‐3607**	0.0	474.1	Inf	< 0.001	0.001
**miR‐125b‐5p**	76.6	894.1	3.5	< 0.001	< 0.001
**miR‐133a‐3p**	0.0	71.7	Inf	< 0.001	0.001
**miR‐1‐3p**	251.3	15323.8	5.9	< 0.001	< 0.001
**miR‐218‐5p**	1.6	205.8	7.0	< 0.001	< 0.001
**miR‐338‐3p**	1.9	109.9	5.9	< 0.001	0.002
**miR‐9‐5p**	39.3	717.7	4.2	< 0.001	< 0.001
**miR‐99a‐5p**	2258.4	6795.9	1.6	< 0.001	0.029

*Note:* Forty‐five miRNAs were found to have statistical differences between malignant and benign SPNs by high‐throughput sequencing, and eight miRNAs shown in the table were further selected. Counts per million (CPM) signals were expressed as means.

### 3.2 Plasma Levels of Candidate Plasma miRNAs in the Identification Set

For large scaled analysis of the eight candidate miRNAs, 77 patients, including 37 cases of pure ground glass nodules, 17 cases of mixed ground glass nodules, and 23 cases of solid nodules, were included in the identification set. Pathological examination of surgically removed tissues indicated 48 patients with malignant SPNs and 29 patients with benign SPNs. Within 48 malignancies, 10 were adenocarcinomas in situ, 22 minimally invasive adenocarcinoma, 15 invasive adenocarcinoma and one adenosquamous carcinoma. In the 29 cases of benign SPNs, 15 were nonspecific inflammation, eight fibroplasia, three tuberculosis, one sclerosing pneumocytoma, one pneumoconiosis, and one organizing pneumonia. Detailed clinicopathological characteristics of the identification set patients with SPN are summarized in Table 2.

**TABLE 2:** Characteristics of patients with SPN in the identification set.

Characteristics	Patients with malignant SPNs (*n* = 48)	Patients with benign SPNs (*n* = 29)	*p*
**Age** (Ave ± SD, years)	56.9 ± 12.8	51.1 ± 11.4	**0.049**
**Sex**: male	15 (31.3%)	13 (44.8%)	0.230
Female	33 (68.7%)	16 (55.2%)	
**Nodule size** (Ave ± SD, mm)	12.4 ± 4.5	9.3 ± 4.5	**0.006**
**Nodule location**			0.193
LUL	5 (10.4%)	5 (17.2%)	
LLL	7 (14.6%)	6 (20.7%)	
RUL	23 (47.9%)	6 (20.7%)	
RML	5 (10.4%)	4 (13.8%)	
RLL	8 (16.7%)	8 (27.6%)	
**Density**			**< 0.001**
Solid	7 (14.6%)	16 (55.2%)	
pGGN	24 (50.0%)	13 (44.8%)	
mGGN	17 (35.4%)	0 (0%)	
**Pathology**			**< 0.001**
AIS	10 (20.8%)		
MIA	22 (45.8%)		
IAC	15 (31.3%)		
ADCA	1 (2.1%)		
Nonspecific inflammation		15 (51.7%)	
Fibroplasia		8 (27.6%)	
Tuberculosis		3 (10.3%)	
Sclerosing pneumocytoma		1 (3.45%)	
Pneumoconiosis		1 (3.45%)	
OP		1 (3.45%)	

*Note:* Age, sex, nodule size, nodule location, and density of patients with malignant and benign SPNs were analyzed by Student's *t*‐test or Chi‐square test.

Abbreviations: ADCA, adenosquamous carcinoma; AIS, adenocarcinoma in situ; IAC, invasive adenocarcinoma; LLL, left lower lobe; LUL, left upper lobe; mGGN, mixed ground glass nodules; MIA, minimally invasive adenocarcinoma; OP, organizing pneumonia; pGGN, pure ground glass nodules; RLL, right lower lobe; RML, right middle lobe; RUL, right upper lobe.

**FIGURE 2:**
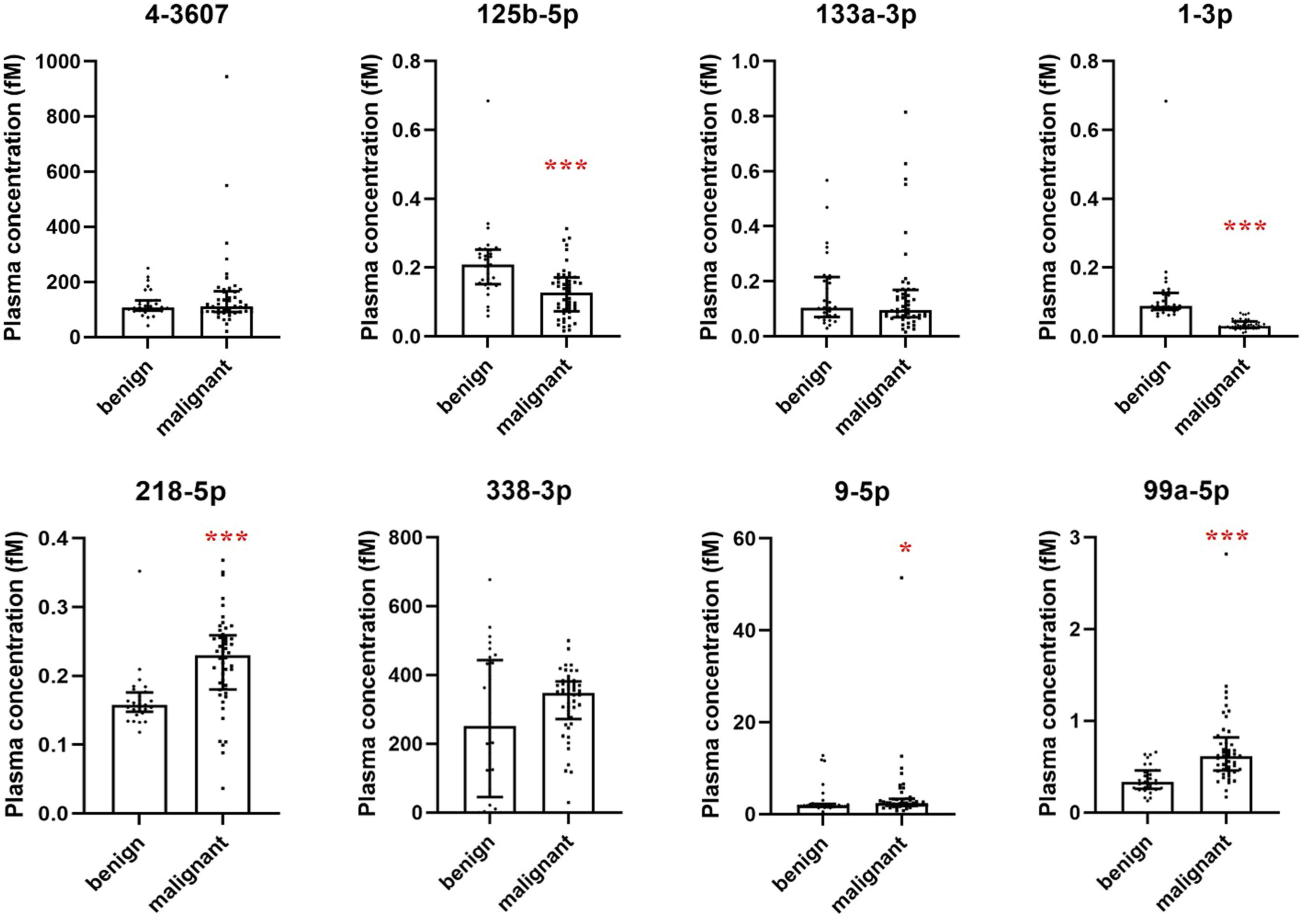
Plasma concentrations of the eight candidate miRNAs in the patients with SPN of the identification set. Plasma concentrations of the candidate miRNAs in 48 patients with malignant and 29 patients with benign SPN in the identification set were detected by qPCR. The Mann–Whitney U test was used for statistical analysis. Horizontal lines indicate median values with interquartile range. * and *** indicate *p* <  0.05 and *p* <  0.001 between the patients with benign and malignant SPN. Abbreviations: fM, ficomoles/L. miR‐4‐3607 is a novel miRNA.

Quantitative PCR analysis revealed that among the eight candidate miRNAs, the plasma levels of five miRNAs (miR‐125b‐5p, miR‐1‐3p, miR‐218‐5p, miR‐99a‐5p, and miR‐9‐5p) were statistically different between benign and malignant SPNs (Figure 2). The median plasma concentrations of miR‐125b‐5p and miR‐1‐3p in the malignant SPNs were significantly lower than those of the benign SPNs, while the plasma levels of miR‐218‐5p, miR‐99a‐5p, and miR‐9‐5p were higher in the malignant SPNs (Figure 2).

### 3.3 Analysis of five miRNAs as Biomarkers for Malignant SPNs in Identification

**FIGURE 3:**
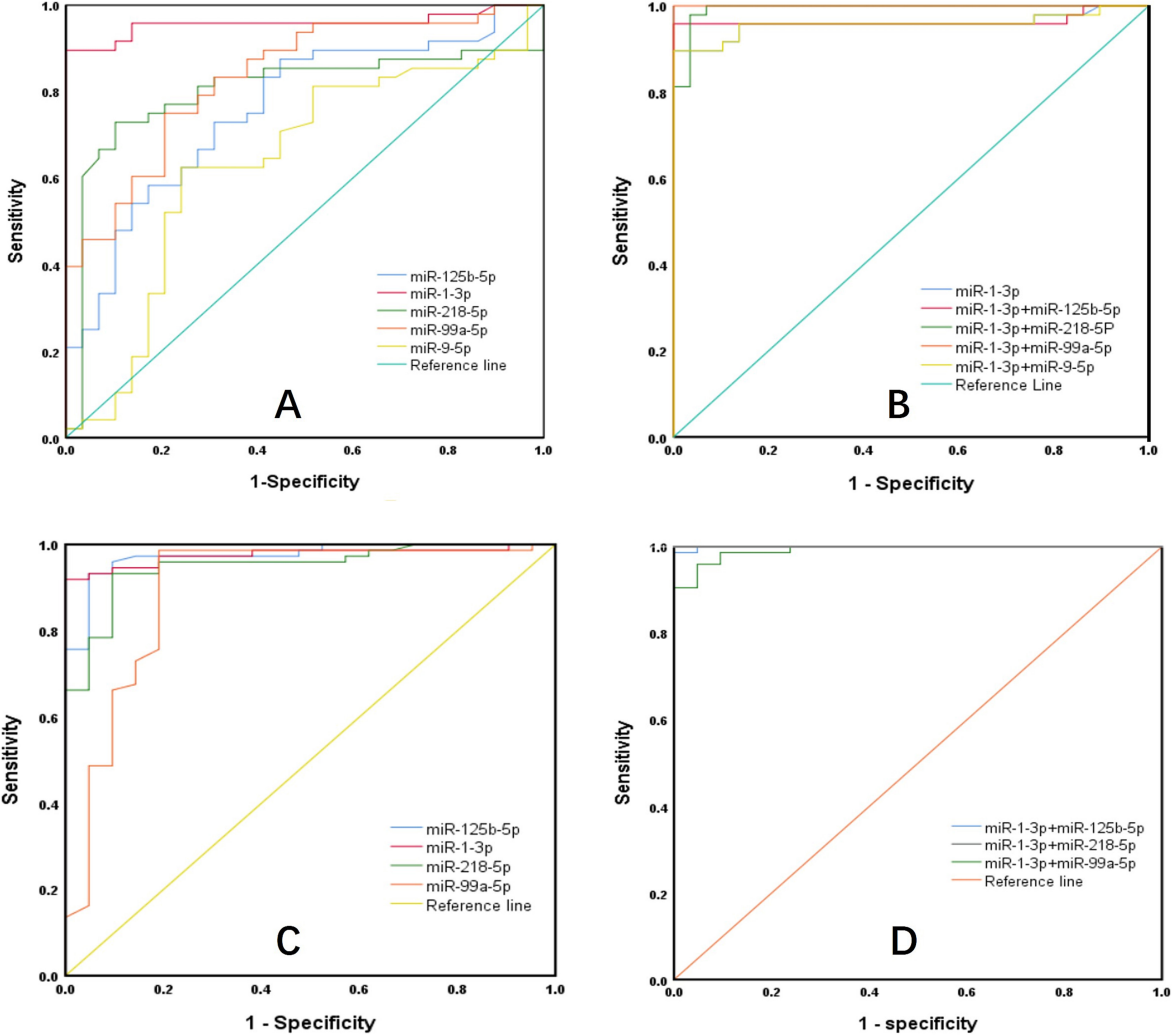
AUROC analysis of miRNAs for malignant SPNs in the identification set and validation set. The miRNAs identified as potential biomarkers for malignant SPNs each alone (A), or combined (B) in the identification and (C), (D) in the validation set, were subjected to area under the receiver operating characteristic curve (AUROC) analysis.

The five plasma miRNAs with statistical differences in the plasma levels were further evaluated as indicators of malignant SPNs by using AUROC analysis. The comparison of two ROC curves was tested by DeLong's test to judge whether there is a difference in their diagnostic value. As shown in Figure 3 and Table 3, the sensitivity and specificity were 87.5% and 55.2% for miR‐125b‐5p, 89.6% and 100% for miR‐1‐3p, 72.9% and 89.7% for miR‐218‐5p, 75% and 79.3% for miR‐99a‐5p, and 62.5% and 75.9% for miR‐9‐5p, respectively. MiR‐1‐3p had the highest sensitivity and specificity and the best diagnostic value (AUC = 0.958) compared with other single miRNA (DeLong's test, all *p* <  0.05), with the critical value ≤ 0.055 ficomoles/L.

**TABLE 3:** Diagnostic values of miRNAs for malignant SPNs in the identification set.

	AUC	95% CI	*p*	Critical value	Sensitivity	Specificity
**miR‐125b‐5p**	0.759	0.650–0.868	< 0.001	≤ 0.197	0.875	0.552
**miR‐1‐3p**	0.958	0.910–1.000	< 0.001	≤ 0.055	0.896	1.000
**miR‐218‐5p**	0.800	0.693–0.908	< 0.001	≥ 0.186	0.729	0.897
**miR‐99a‐5p**	0.834	0.744–0.924	< 0.001	≥ 0.464	0.750	0.793
**miR‐9‐5p**	0.637	0.504–0.770	0.045	≥ 2.150	0.625	0.759
**1‐3p+125b‐5p**	0.965	0.917–1.000	< 0.001	≤ 0.484	0.958	1.000
**1‐3p+218‐5p**	0.993	0.979–1.000	< 0.001	≤ 0.397	0.979	0.966
**1‐3p+99a‐5p**	1.000	1.000–1.000	< 0.001	≤ 0.500	1.000	1.000
**1‐3p+9‐5p**	0.958	0.909–1.000	< 0.001	≤ 0.678	0.896	1.000

The miRNAs each alone, or combined, were subjected to area under the receiver operating characteristic curve analysis for malignant SPNs. The combinations of miRNAs were established with Binary logistic regression. The equations are as follow: Probability of malignancy = e^α^/ (1 + e^α^); For “miR‐1‐3p + miR‐125b‐5p”, α = 67.02 * (1‐3p) + 3.64 * (125b‐5p)‐5.227; For “miR‐1‐3p + miR‐218‐5p”, α = 94.54 * (1‐3p)‐33.81 * (218‐5p)‐0.397; For “miR‐1‐3p + miR‐99a‐5p”, α = 224.9 * (1‐3p) ‐31.59 * (99a‐5p)‐0.353; For “miR‐1‐3p + miR‐9‐5p”, α = 71.61 * (1‐3p)‐0.08 * (9‐5p)‐4.716.

Abbreviations: AUC, area under the curve; CI, confidence interval.

To further improve the diagnostic ability, we combined miR‐1‐3p with each of the other four miRNAs to find out the best diagnostic performance. Binary logistic regression was used to construct different miRNA combination prediction models in the identification set. The regression equation was shown in Table 3.Unexpectedly, when miR‐1‐3p was combined with miR‐99a‐5p, both of the sensitivity and specificity reached 100%, with the AUC of 1.00. MiR‐1‐3p combined with miR‐125b‐5p, miR‐218‐5p, or miR‐9‐5p increased the sensitivities and specificities to 95.8% and 100%, 97.9% and 96.6%, 89.6%, and 100%, respectively. These results indicate that the three combinations (miR‐1‐3p+miR‐99a‐5p, miR‐1‐3p + miR‐125b‐5p and miR‐1‐3p+miR‐218‐5p) could be used as sensitive and specific biomarkers for distinguishing malignant from benign SPNs. Compared with single miR‐1‐3p, the combinations of miRNAs have better diagnostic accuracy tested by DeLong's test (all *p* <  0.01).

### 3.4 Evaluation of the Prediction Values of the Panels of miRNAs in Differentiating Malignant From Benign SPNs in the Validation Set

For further evaluation of the prediction values of the three microRNA combinations (miR‐1‐3p+miR‐99a‐5p, miR‐1‐3p+miR‐125b‐5p and miR‐1‐3p+miR‐218‐5p), 95 patients diagnosed as SPN by CT examination, designated as the validation set, were enrolled for detection of the plasma exosomal miRNAs and for their diagnostic value in a blinded fashion using the optimal critical values established in the above identification set. Clinicopathological information of the 95 patients with SPN (74 patients with malignant and 21 patients with benign SPNs determined by pathological examination of the surgically removed SPN tissues) was summarized in Table 4.

**TABLE 4:** Characteristics of patients with SPN in the validation set.

Characteristics	Patients with malignant SPNs (*n* = 74)	Patients with benign SPNs (*n* = 21)	*p*
**Age** (Ave ± SD, years)	56.9 ± 12.5	54.5 ± 11.5	0.514
**Sex**: male	35 (47.3%)	13 (61.9%)	0.237
Female	39 (52.7%)	8 (38.1%)	
**Nodule size** (Ave ± SD, mm)	13.5 ± 5.6	14.7 ± 7.4	0.497
**Nodule location**			0.189
LUL	17 (23.0%)	5 (23.8%)	
LLL	13 (17.6%)	2 (9.5%)	
RUL	26 (35.1%)	3 (14.3%)	
RML	7 (9.5%)	2 (9.5%)	
RLL	11 (14.8%)	9 (42.9%)	
**Density**			< 0.001
Solid	16 (14.6%)	14 (66.7%)	
pGGN	43 (50.0%)	5 (23.8%)	
mGGN	15 (35.4%)	2 (9.5%)	
**Pathology**			
AIS	32 (43.2%)		
MIA	22 (29.7%)		
IAC	16 (21.6%)		
SCC	3 (4.1%)		
SCLC	1 (1.4%)		
Nonspecific inflammation		11 (52.4%)	
Fibroplasia		2 (9.5%)	
Tuberculosis		1 (4.8%)	
Sclerosing pneumocytoma		1 (4.8%)	
Hamartoma		4 (18.9%)	
Cryptococcal infection		1 (4.8%)	
OP		1 (4.8%)	

*Note:* Age, sex, nodule size, nodule location and density of patients with malignant and benign SPNs were analyzed by Student's *t*‐test or Chi‐square test.

Abbreviations: AIS, adenocarcinoma in situ; IAC, invasive adenocarcinoma; LLL, left lower lobe; LUL, left upper lobe; mGGN, mixed ground glass nodules; MIA, minimally invasive adenocarcinoma; OP, organizing pneumonia; pGGN, pure ground glass nodules; RLL, right lower lobe; RML, right middle lobe; RUL, right upper lobe; SCC, squamous cancer; SCLC, small cell lung cancer.

**FIGURE 4:**
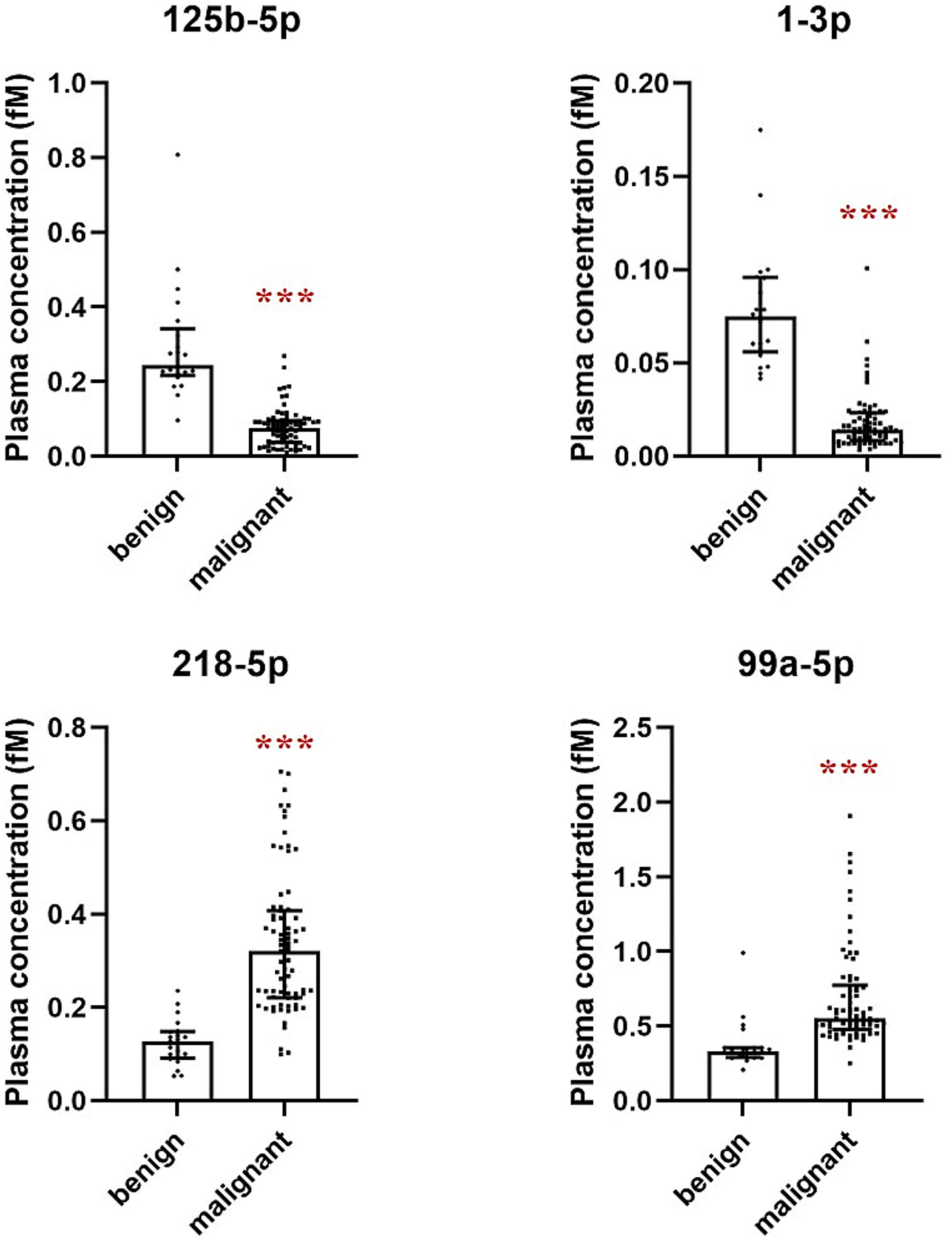
Plasma concentrations of the four miRNAs in the patients with SPN of the validation set. Plasma concentrations of the four miRNAs in 74 patients with malignant and 21 patients with benign SPN were detected by qPCR. The Mann–Whitney U test was used for statistical analysis. Horizontal lines indicate median values with interquartile range. *** indicates *p* <  0.001 between the patients with benign and malignant SPN. Abbreviations: fM, ficomoles/L.

As in the validation set, the plasma levels of all the four miRNAs also had statistical differences between the malignant SPNs group with the benign SPNs (Figure 4). AUROC analysis indicated that in all the three combinations, miR‐1‐3p + miR‐218‐5p had the best prediction performance with a positive predictive value (PPV) of 97.4% and a negative predictive value (NPV) of 100%, and its sensitivity and specificity reached 100% and 90.5%, respectively (Table 5 and Figure 3). Although the other two combinations also had relatively high PPV, NPV, and sensitivity, the specificities were less than 80%. In addition, combinations of three or four miRNAs did not enhance the diagnostic performance (data not shown).

**TABLE 5:** Diagnostic value of miRNAs and their combinations in the validation set.

	AUC	95% CI	Critical value	Sensitivity	Specificity	PPV	NPV
**miR‐125b‐5p**	0.974	0.944–1.00	≤ 0.197	0.973	0.810	0.947	0.895
**miR‐1‐3p**	0.976	0.947–1.00	≤ 0.055	0.973	0.762	0.935	0.889
**miR‐218‐5p**	0.949	0.904–0.995	≥ 0.186	0.932	0.857	0.958	0.783
**miR‐99a‐5p**	0.897	0.802–0.992	≥ 0.464	0.797	0.810	0.937	0.531
**1‐3p+125b‐5p**	1.00	1.00–1.00	≤ 0.484	0.973	0.762	0.935	0.889
**1‐3p+218‐5p**	1.00	1.00–1.00	≤ 0.397	1.00	0.905	0.974	1.00
**1‐3p+99a‐5p**	0.993	0.982–1.00	≤ 0.500	1.00	0.667	0.914	1.00

*Note:* The miRNAs each alone, or combined, were subjected to area under the receiver operating characteristic curve analysis for malignant SPNs.

Abbreviations: AUC, area under the curve; CI, confidence interval; PPV, positive predictive value; NPV, negative predictive value.

## 4 Discussion

SPNs become increasingly frequent as CT is broadly applied in early lung cancer screening. Due to their high etiological diversity and potential for malignancy, rapid identification and subsequent resection of malignant SPNs are crucial in the clinical management. Currently, CT examination is still a major method for determining that an SPN is malignant or not. Characteristics in CT imaging, together with risk factors such as age and smoking history, are the basis for the judgement. The nodules size is positively correlated with the lung cancer diagnosis rate, and 5% are found to be malignant in nodules smaller than 10 mm in diameter, 21.3% for nodules between 11–19 mm, and 34.5% for nodules larger than 20 mm [19]. Other features of CT imaging including lobar location, density and margin characteristics are helpful for the diagnosis [20, 21, 22]. Unfortunately, there are still 30% to 50% of the nodules found to be benign after the surgical operation, leading to a high false positive rate or overdiagnosis [6].

Liquid biopsies, referring to detection of tumor cells and tumor‐derived products in body fluids, have made a great progress in cancer diagnosis in recent years [23]. Compared with traditional tissue biopsies in which surgery and other invasive operations are needed, liquid biopsies have the advantages of non‐invasive and sustainable application, making it possible to monitor the changes of tumor molecules in real time [24]. Lung cancer‐derived exosomal miRNAs are key regulators in intercellular communication of tumor microenvironment and play regulatory roles in the development of lung cancer through the interaction with receptor cells. It has been reported that exosomal miR‐21 upregulates the expression of vascular endothelial growth factor receptor by activating STAT3, and eventually induce angiogenesis and malignant transformation of human bronchial epithelial cells [25]. Similarly, exosmal miR‐494 and miR‐542‐3p from pulmonary interstitial cells down‐regulate the expression of cadherin‐17, thus increasing the expression level of matrix metalloproteinase MMP2 and MMP3 and promoting the distant metastasis of lung cancer cells [12]. Meanwhile, circulating exosomal miRNAs in the plasma or serum as a diagnostic and/or prognostic biomarker have also been investigated in recent clinical studies [14, 15, 16, 17, 18]. Although some exosomal miRNAs have potentials as lung cancer diagnosis biomarkers, their abilities in the diagnosis of malignant SPNs remain limited, especially for small SPNs, owing to not satisfactory sensitivities and specificities.

In the current study, 77 patients with small SPN, with the average nodule sizes of 12.4 mm and 9.3 mm for malignant and benign SPN, respectively, were enrolled in the identification set. Quantitative PCR detection of eight candidate miRNAs screened and selected by high throughput sequencing in the identification set showed that five plasma exosomal miRNAs (miR‐125b‐5p, miR‐1‐3p, miR‐218‐5p, miR‐9‐5p and miR‐99a‐5p) were identified as potential biomarkers for malignant SPNs. miR‐1‐3p single alone had the best diagnostic performance with the sensitivity of 89.6% and specificity of 100%. When it was combined with miR‐125b‐5p, miR‐218‐5p, or miR‐99a‐5p, both the specificity and sensitivity were increased to more than 95%, indicating the combinations were able to effectively differentiate malignant from benign SPNs. For further observation of prediction values of the miRNA combinations, 95 patients with SPN were enrolled in the validation set. Blinded detection of the miRNAs and analysis of the diagnostic reliability of the miRNA combinations by using the optimal critical values established in the identification set revealed that miR‐1‐3p combined with miR‐218‐5p, miR‐99a‐5p or miR‐125b‐5p had a good prediction performance with a positive predictive value (PPV) over 90% and a negative predictive value (NPV) of 100%, 100% and 88.9%. In brief, our results indicate that the three combinations are sensitive and specific biomarkers for malignant SPNs.

MiR‐1‐3p has been reported to be implicated in a variety of cancer development and progression by targeting different proteins in different cellular contexts [26, 27, 28]. MiR‐99a‐5p down‐regulates CDC25A to inhibit breast cancer progression [29] and can serve as a biomarker for hepatocellular carcinoma [30]. MiR‐218‐5p is associated with pathogenesis of chronic obstructive pulmonary disease [31] and capable of suppressing retinoblastoma progression [32]. MiR‐125b‐5p is involved in tumorigenesis and progression of lung adenocarcinoma and pancreatic cancer [33, 34]. Since the miRNAs identified in our study are extensively expressed, it is likely that the miRNAs are not specific for lung cancer development. Combined detection of the miRNAs as biomarkers for malignant SPN will elevate the specificity as well as the sensitivity. However, their roles in the lung carcinogenesis and origins need further study.

A recent meta‐analysis including 14 publications and 17 studies about the diagnostic value of circulating miRNA in benign and malignant pulmonary nodules showed that the pooled sensitivity for miRNA in diagnosing benign and malignant pulmonary nodules was 82% and specificity was 84%. However, most of the studies are small sample studies with certain limitations, including lack of the miRNA screening procedure and the constructing diagnostic criteria and diagnostic thresholds differed between studies and have not been reported in around half of the literature [35]. Compared with previous studies, our results showed higher sensitivity and specificity, which may be due to the methods that we use miRNAs from plasma exosomes rather than free miRNAs in blood, which may enable us to obtain more accurate results. In addition, we adopted high‐throughput sequencing for the selection of miRNAs, which makes it possible to find new miRNAs for follow‐up studies.

It should also be noted that, although these miRNA combinations seem to be sensitive and specific biomarkers for identification of malignant SPNs, the sample size was small in our current study and the patients with SPN were enrolled in only one medical center. These limitations may lead to a potential bias. Furthermore, the pulmonary nodule size in our study is about 1 cm, and the diagnostic value for smaller nodules still needs to be verified. In addition, it is strange that expression of miR‐1‐3p and miR‐125b‐5p analyzed by high‐throughput sequencing was higher in patients with malignant SPN than patients with benign SPN, while qPCR detection in the identification and validation sets showed contrary results. This is possibly ascribed to different methods used.

## 5 Conclusion

Through high throughput sequencing, qPCR determination of plasma microRNAs and AUROC analysis, miR‐1‐3p combined with miR‐99a‐5p, miR‐125b‐5p, or miR‐218‐5p have been found to be sensitive and specific indicators of malignant SPNs in both the identification and validation set. Our results indicate that the panels of plasma miRNAs are potential diagnostic biomarkers for malignant SPNs.

## Author Contributions

RT, JX, JY and DZ designed the research. RT, DW, WP, YL and PL collected clinical samples and data. RT, RL, JX and JY performed experiments. RT, JX and DZ collected, analyzed and interpreted data. RT, DZ and JX wrote the manuscript.

## Ethics Statement

This study was approved by Ethics Committee of the Second Affiliated Hospital of Anhui Medical University (approval number is YX2019‐046(F1)).

## Conflicts of Interest

Panels of miRNAs, i.e., miR‐1‐3p and miR‐99a‐5p, miR‐1‐3p and miR‐218‐5p, and miR‐1‐3p and miR‐125b‐5p, are applying national invention patents (Application numbers are 202111530540.7 and 202 111 530 337.X).

## Acknowledgements

This study was supported by the Hefei Municipal Natural Science Foundation (2021037), the Collaborative Chinese and Western Medicine Research Project for Major Difficult Diseases (2021zdynjb06), the Natural Science Research Project of Anhui Universities (KJ2021ZD0028), the Research Fund of Anhui Institute of Translational Medicine (2021zhyx‐c67) and the Anhui Province Natural Science Foundation (Grant No. 2208085MH194). We are grateful to Dr. Faming Pan for his kind statistics checking.

## Data Availability

The data that support the findings of this study are available on request from the corresponding author. The data are not publicly available due to privacy or ethical restrictions.
